# Urban-rural disparity in stunting among Ethiopian children aged 6–59 months old: A multivariate decomposition analysis of 2019 Mini-EDHS

**DOI:** 10.1371/journal.pone.0284382

**Published:** 2023-04-19

**Authors:** Sisay Eshete Tadesse, Tefera Chane Mekonnen, Reta Dewau, Aregash Abebayehu Zerga, Natnael Kebede, Yitbarek Wasihun Feleke, Amare Muche

**Affiliations:** 1 Department of Nutrition and Dietetics, School of Public Health, College of Medicine and Health Sciences, Wollo University, Dessie, Ethiopia; 2 Department of Epidemiology and Biostatistics, School of Public Health, College of Medicine and Health Sciences, Wollo University, Dessie, Ethiopia; 3 Department of Health Behaviour and Communication, School of Public Health, College of Medicine and Health Sciences, Wollo University, Dessie, Ethiopia; National Research Centre, EGYPT

## Abstract

**Background:**

Childhood stunting is still a global public health challenge, including in Ethiopia. Over the past decade, in developing countries, stunting has been characterized by large rural and urban disparities. To design an effective intervention, it is necessary to understand the urban and rural disparities in stunting.

**Objective:**

To assess the urban-rural disparities in stunting among Ethiopian children aged 6–59 months.

**Methods:**

This study was done based on the data obtained from the 2019 mini-Ethiopian Demographic and Health Survey, conducted by the Central Statistical Agency of Ethiopia and ICF international. The result of descriptive statistics was reported using the mean with standard deviation, frequency, percentages, graphs, and tables. A multivariate decomposition analysis was used to decompose the urban-rural disparity in stunting into two components: one that is explained by residence differences in the level of the determinants (covariate effects), and the other component is explained by differences in the effect of the covariates on the outcome (coefficient effects). The results were robust to the different decomposition weighting schemes.

**Result:**

The prevalence of stunting among Ethiopian children aged 6–59 months was 37.8% (95% CI: 36.8%, 39.6%). The difference in stunting prevalence between urban and rural residences was high (rural prevalence was 41.5%, while in urban areas it was 25.5%). Endowment and coefficient factors explained the urban-rural disparity in stunting with magnitudes of 35.26% and 64.74%, respectively. Maternal educational status, sex, and age of children were the determinants of the urban-rural disparity in stunting.

**Conclusion and recommendation:**

There is a significant stunting disparity among urban and rural children in Ethiopia. A larger portion of the urban-rural stunting disparity was explained by coefficient effects (differences in behaviour). Maternal educational status, sex, and age of children were the determinants of the disparity. So, to narrow this disparity, emphasis should be given to both resource distribution and the appropriate utilization of available interventions, including improvement of maternal education and consideration of sex and age differences during child feeding practices.

## Introduction

Stunting is defined as having a height-for-age Z score that is 2 standard deviations below the median child growth standard as described by the world health organization (WHO) [1]. Worldwide, nearly 21.9% of under-five children were having stunted growth in 2020 [2]. Only 27.8% of the world’s countries are on track to reduce stunting by 2025 [3]. Early childhood stunting has been linked with poor cognitive and academic performance, poor health outcomes, loss of productivity, and a higher risk of chronic diseases and impairments connected to inadequate nutrition [1, 4, 5].

Lack of protein and energy, poor maternal nutritional status, insufficient consumption of animal-source foods, intrauterine growth restriction, improper child feeding practices, recurrent infections, food insecurity, water sanitation and hygiene, socio-demographic factors, and economic factors are all the contributor to the occurrence of stunted growth [6–8]. The first 1000 days are the most critical and suitable time for creating a generation free of stunting [9]. In order to sustain the quick growth and development, prevent infections, and lessen susceptibility to biological programming, there are heightened dietary needs during this period [9, 10].

Despite the exertions made by all the concerned stakeholders, stunting is still a public health challenge in Ethiopia [11]. The current rate of progress is not fast enough to achieve the 2025 target for stunting reduction [9]. Childhood stunting in non-industrialized nations has been characterized by large urban-rural disparities over the last few years [12–14]. There is limited evidence on urban-rural disparities in stunting among children based on nationally representative data using multivariate decomposition analysis [15]. Therefore, the purpose of this study was to assess the urban-rural disparities in stunting among children aged 6–59 months using the 2019 mini-Demographic and Health Survey (MEDHS). This study will help health practitioners and policymakers understand the extent and causes of disparity in order to design and plan appropriate nutrition interventions.

## Methods and materials

### Data source

This finding was based on the 2019 MEDHS dataset. The data were obtained in STATA format from the website http://dhspr/ogram.com/data/. The required variables for the study were extracted from the Integrated Public Use Microdata Series (IPUMS) of the DHS (Demographic and Health Survey) [16].

### Study design and period

A community based cross sectional study was conducted from March 21, 2019 to June 28, 2019.

### Source population

The source population was all mother/care-givers with their children in the age range of 6–59 months in Ethiopia.

### Sampled population

The sampled population was all mother/care-givers with their children in the age range of 6–59 months in each household in the enumeration area.

### Sample size

This study included 643 clusters and 4,292 children aged 6–59 months old.

### Sampling procedure

Twenty one sampling strata were produced after stratifying each region into urban and rural areas. In each stratum, samples from the enumeration areas (EAs) were selected independently in two stages. In the first stage, a total of 305 EAs (93 from urban and 212 from rural areas) were selected proportional to the size of the EA (based on the 2019 PHC frame) and with independent selection in each sampling stratum. A household listing operation was carried out in all selected EAs from January to April, 2019. The resulting lists of households served as a sampling frame for the selection of households in the second stage. Some of the EAs selected for the 2019 MEDHS were large, with more than 300 households. To minimize the task of household listing, each large EA selected for the 2019 MEDHS was segmented. Only one segment was selected for the survey, with probability proportional to the segment size. Household listing was conducted only in the selected segment.

In the second stage of selection, a fixed number of 30 households per cluster were selected with an equal probability through systematic selection from the newly created household listing. All children aged 6 to 59 months who were either permanent residents or visitors and slept in the household the night before the survey were eligible for an interview.

### Data collection tool

Face-to-face interviews with mother/care-givers was used to collect data. In all households, height and weight measurements were recorded for all children aged 6–59 months. Weight measurements were obtained using lightweight, electronic SECA 874 scales with a digital screen. Height measurements were carried out with measuring boards donated by the United Nations International Children’s Emergency Fund (UNICEF). Children younger than 24 months of age were measured in a recumbent position on the board, while standing height was measured for older children. In contrast with the data collection procedures for household and individual interviews, anthropometry data were initially recorded on the paper-based Biomarker Questionnaire and subsequently entered into interviewers’ tablet computers.

### Data analysis

The data were analysed by STATA version 14.0. Weighted frequencies and percentages were calculated to account for DHS and design effects. The result of descriptive statistics was reported as the mean with standard deviation, frequency, percentages, a graph, and tables. A multivariate decomposition analysis was used to decompose the urban-rural disparity in stunting. Multivariate decomposition uses the regression models’ output, such as a mean or proportion, to partition into a component attributable to compositional differences between groups. This technique decomposes the urban-rural gap in stunting into the endowment effect (the contribution of respondents’ characteristics and their environment) and the coefficient effect (the contribution of response to behaviour).

As a result, the difference in stunting can be attributed to either a gap in endowments (E), the coefficients (C), or the interaction of endowments and coefficients. The multivariate decomposition considers the high group (urban children in this study) as the reference group, weighting contrasts in attributes by the coefficients of urban children and contrasts in coefficients by the covariates of rural children [17]. A coefficient with a 95% confidence interval and a p value of 0.05 were used to declare the statistical significance.

### Ethical clearance

Before data collection, MEDHS data collection materials were approved for compliance with the requirements of “Protection of Human Subjects” by the Institutional Review Board (IRB) of the country. Written informed consent was obtained from study participants. Participants were informed that participation was on a voluntary basis. The survey data were received from the DHS International Program upon submission of a proposal. Confidentiality was maintained after data access was authorized by DHS.

## Results

### Socio-demographic characteristics of study participants

A total of 4,292 children were included in the study. The mean age of mothers/care-givers was 28.9±0.1 (standard deviation) years. More than half 62.4% of rural residents and 30.3% of urban residents couldn’t read and write. In both urban and rural settings, more than half of children aged 6–59 months were male. More than two-thirds (68.4%) of children in rural areas and 61.2% of children in urban areas were found in the age range of 24–59 months old. Regarding the wealth index distribution, almost 61% and 41% of rural and urban residents were below the poverty line, respectively. Half (50.9%) of rural residents and 42.1% of urban residents were from an agrarian region. Almost all of the study participants from both rural and urban settings had a family size of less than or equal to 3 (Table 1).

**Table 1:** Socio-demographic characteristics of study participants based on place of residence using 2019 Mini-Ethiopian Demographic and Health Survey, 2022.

Variables		Urban	Rural	Remark
Maternal age (Mean±SD) 28.9±0.1				
Educational status of mother	Cannot read and write	303 (30.3%)	2053 (62.4%)	
	Primary	336 (33.6%)	1004 (30.5%)	
	Secondary and higher	360 (36.1%)	236 (7.1%)	
Sex of Child	Male	502 (50.3%)	1697 (51.6%)	
	Female	497 (49.7%)	1595 (48.4%)	
Age of Child	6–23 month	368 (36.8%)	1042 (31.6%)	
	24–59 months	631 (63.2%)	2251 (68.4%)	
Wealth Index	Poor	109 (10.4%)	2176 (63.1%)	
	Middle	31 (2.9%)	606 (17.6%)	
	Rich	909 (86.7%)	667 (19.3%)	
Marital Status	Married	169 (92.9%)	1152 (93.8%)	
	Not Married	13 (7.1%)	76 (6.2%)	
Region	Agrarian	80 (42.1%)	623 (50.9%)	
	Pastoralist	74 (41%)	555 (45%)	
	Urban	28 (16.9%)	50 (4.1%)	
Number of Under five children	≤3	177 (97.2%)	1198 (97.6%)	
	4–5	5 (2.8%)	30 (2.4%)	

### Maternal and child-related characteristics

More than three-fourths (88.7%) of the study participants from urban areas and 62% of women from rural areas have access to antenatal care services. Home delivery is still higher among rural residents, and postnatal care service utilization was lower in both urban and rural study participants. More than half of rural children and 36.6% of urban children were not vaccinated. Nearly 82% of urban children and 81% of rural children initiated breastfeeding within an hour after delivery. Prelacteal feeding is still practiced in Ethiopia (Table 2).

**Table 2:** Health care and child feeding related characteristics of study participants based on place of residence using 2019 Mini-Ethiopian Demographic and Health Survey, 2022.

Variables		Urban	Rural	Remark
Antenatal Care	Yes	709 (88.7%)	1563 (62%)	
	No	69 (11.3%)	682 (38%)	
Place of delivery	health facility	837 (84.3%)	1295 (39.9%)	
	Home	156 (15.7%)	1951 (60.1%)	
Postnatal Care	Yes	32 (15.1%)	129 (9.7%)	
	No	180 (84.9%)	1203 (90.3)	
Vaccination	Yes	115 (63.4%)	533 (43.4%)	
	No	67 (36.6%)	695 (56.6%)	
Duration of breastfeeding	Ever Breastfed	622 (62.6%)	2023(62.3%)	
	Still Breastfed	371(37.4%)	1,223 (37.7%)	
Initiation of Breastfeeding	Within an hour	530 (76.4%)	1762 (72.4%)	
	After a hour	127 (18.3%)	550 (22.6%)	
	After 24 days	37 (5.3%)	121 (5%)	
Prelacteal feeding	Yes	28 (14%)	244 (19.1%)	
	No	172 (81.1%)	1035 (80.9%)	
Exclusive breastfeeding	Yes	9 (4.5%)	98 (8%)	
	No	173 (95.5%)	1130 (92%)	
Vitamin A supplement	Yes	313 (54.6%)	759 (44%)	
	No	260 (45.4%)	966 (56%)	
Iron Folic Acid intake	Yes	548 (74.1%)	1162 (56%)	
	No	192 (25.9%)	914 (44%)	
Number of living children	<3	855 (87%)	2637 (82.8%)	
	≥3	128(13%)	546 (17.2%)	

### Urban-rural disparities in stunting among children aged 6–59 months

**Fig 1:**
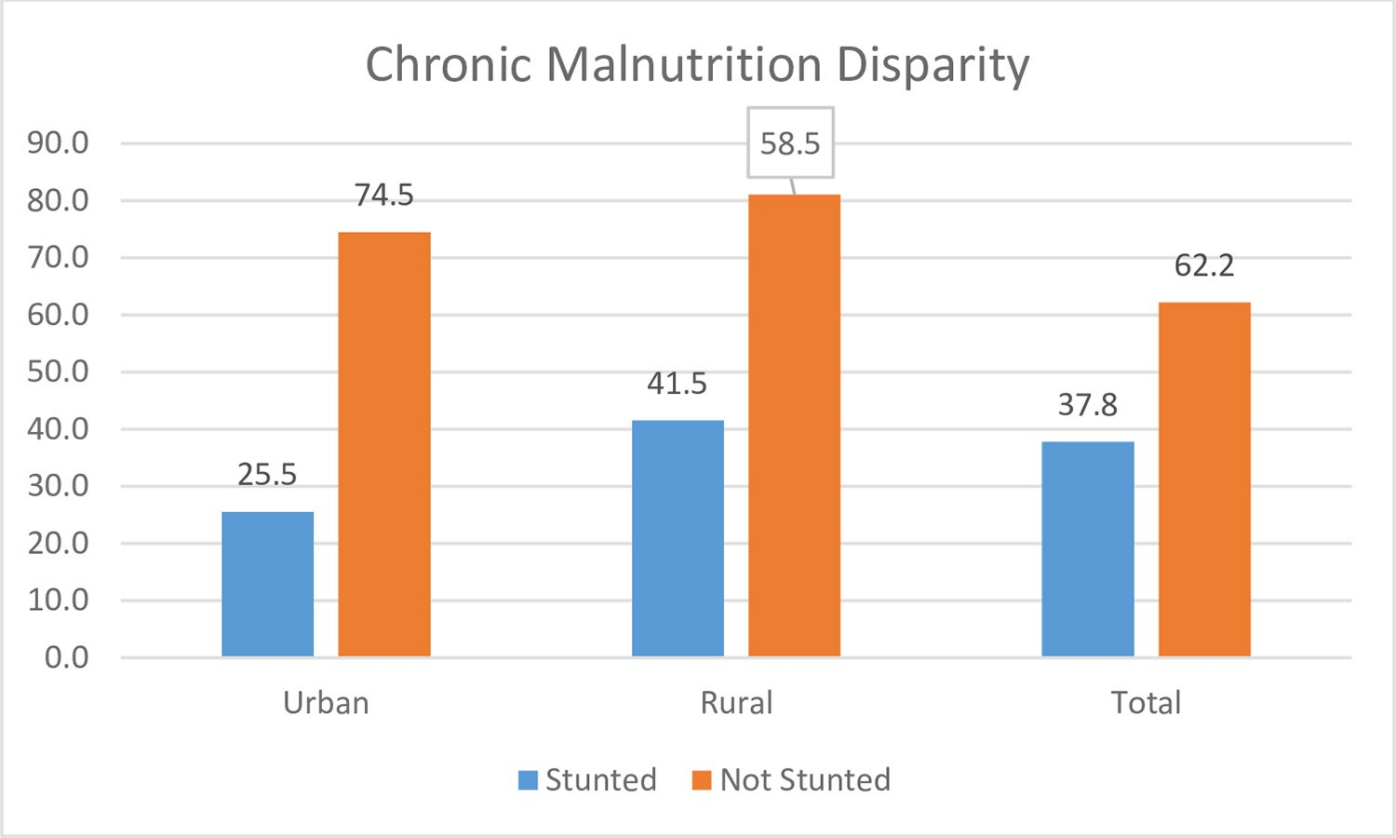
Urban and rural disparities of stunting among Ethiopian children aged 6–59 months, using 2019 mini-EDHS data, 2022.

The prevalence of stunting among children aged 6–59 months was 37.8% (95% CI: 36.8%, 39.6%). The difference in the prevalence of stunting between urban and rural areas was high (rural prevalence was 41.5%, while in urban areas it was 25.5%) (Fig 1).

### Decomposition result

The detail decomposition result showed that there is a significant disparity in stunting between urban and rural residences (0.14, p < 0.001). The difference in stunting between urban and rural households was explained by a component, which accounted for 35.26 percent, and 64.74 percent was supplied by an unaccounted-for component (difference in behaviour). The coefficients effects (difference in behaviour) accounted for 64.74% of the observed residence differences in the prevalence of stunting, with high intercept differences (0.092, p = 0.008).

Shifting rural male distribution to urban levels, would be expected to decrease the urban-rural gap by 0.81%. If rural children aged 24–59 months were not protected from risks to the same degree as urban children, the urban-rural stunting gap would be expected to increase by 1.63%. Controlling for other factors, maternal education would provide a 30.44% reduction in the urban-rural stunting disparity. That is, if rural women received secondary and higher education at the same rate as urban women, the urban-rural disparity would decrease by 30.44% (Table 3) [17].

**Table 3:** Detail decomposition of stunting by place of residence among Ethiopian under five children using 2019 Mini-Ethiopian Demographic and Health Survey, 2022.

**Decomposition**	**Coefficient with 95% CI**		**Percent Explained**	**P-value**
Raw Difference	.142 (0.09, 0.19)		100	0.000
Explained	.0500 (0.001, 0.09)		35.26	0.046
Unexplained	.092 (0.02, 0.16)		64.74	0.008
**Endowment (Explained component) = Difference in characteristics (E)**				
		**Coefficient with 95% CI**	**Percent**	**P value**
Sex of child	Male	0.001 (0.00, 0.002)	0.81	0.004
	Female	1	1	
Age of child	6–23 month	1	1	
	24–59 months	-0.002 (-0.003, -0.001)	-1.63	0.000
Currently on Breastfeeding	Yes	-0.001 (-0.003, 0.002)	-0.47	0.59
	No	1	1	
Wealth Index	Poor	1	1	
	Medium	0.004 (-0.01, 0.02)	3.21	0.58
	Rich	0.02 (-0.022, 0.069)	16.27	0.32
Maternal Educational Status	Cannot read & write	1	1	
	Primary	-0.003(-0.018, 0.006)	-4.38	0.320
	Secondary and Higher	0.04 (0.003, 0.083)	30.44	0.035
Family Size	< = 3	1	1	
	>4	-0.001 (-0.0022, 0.0005)	-0.598	0.212
**Unexplained (Due to difference in coefficients (C))**				
Sex of Child	Male	0.05 (-0.02, 0.101)	34.71	0.06
	Female	1	1	
Age of child	6–23 month	1	1	
	24–59 months	0.34 (-0.073, 0.141)	24.13	0.53
Currently on Breastfeeding	Yes	-.064 (-.204, 0.076)	-45.12	0.37
	No	1	1	
Wealth index	Poor	1	1	
	Middle	0.002 (-0.007, 0.001)	1.59	0.63
	Rich	-0.033 (-0.202, 0.137)	-22.93	0.71
Maternal Educational Status	Illiterate	1	1	
	Primary	0.009 (-0.051, 0.055)	1.32	0.944
	Secondary and Higher	-0.014 (-0.111, 0.082)	-10.18	0.77
Family Size	< = 3	1	1	
	>4	-.0598 (-0.208, 0.089)	-42.294	0.429

N.B: A negative (-) coefficient indicates an increase in the gap, while a positive (+) coefficient indicates the expected decrease in the coefficient.

## Discussion

The aim of this study was to assess the urban-rural disparities in stunting among Ethiopian children aged 6–59 months using the 2019 MEDHS. The result of this study showed that stunting among children in urban areas of Ethiopia was better than that of rural residents. The prevalence of stunting among children aged 6–59 months was 37.8%. Nearly three-fourths of the observed urban-rural stunting disparities among children could be attributed to differences in effect. This implies that the urban-rural gap in stunting would be reduced more by changes in behavioral (effect) than resource distribution (endowments). This finding is consistent with studies conducted in low- and middle-income countries, which found a significant difference in stunting between urban and rural children [12–14, 18]. This might be because urban mothers are capable of getting better information on child feeding practices than rural mothers.

The findings of this study showed that maternal education was an important predictor for narrowing the urban-rural disparities in childhood stunting. Equalizing rural women’s educational status to that of urban women at the secondary and higher educational levels would decrease the urban-rural disparity in stunting.This finding is consistent with studies conducted in Tanzania, South Asia, and Nigeria [19–21]. This might be because mothers with higher educational levels in urban areas have better access to child feeding practices and the utilization of improved healthcare services, which in turn affect health-related choices that improve the nutritional status of children [22].

This study revealed that shifting rural male distribution to urban levels would be expected to decrease the urban-rural stunting gap. This result is concurrent with a finding reported from a meta-analysis of sixteen demographic and health surveys of ten countries in sub-Saharan Africa, in which male children were found to be consistently more likely to become stunted compared to their counterparts [23–25]. This could be because males are more influenced by environmental stress than females [26]. It could also be explained by the motivation of mothers to initiate early complementary feeding for males when they are born small due to pre-existing nutritional status [27, 28].

The gap in stunting is also explained by the age of children. If rural children aged 24–59 months were not protected from risks to the same degree as urban children, the urban-rural stunting gap would increase. This finding is similar to that of previous studies conducted in Bangladesh, Tanzania, Zambia, and Malawi [29–32]. This could be partially elucidated by the protective effect of optimal child feeding practices [19]. It might also be partly explained by the fact that most urban children have easier access to a diversified diet, which contains essential nutrients, than rural kids.

To conclude, there was a significant urban-rural disparity in stunting among children in Ethiopia. A large portion of the urban-rural disparity in stunting was explained by a coefficients (differences in behavior). Maternal educational status, sex, and age of children were the determinants of the urban-rural disparity in stunting. To narrow the urban-rural stunting disparity, emphasis should be given to both resource distribution and the appropriate utilization of available interventions, including improvement of maternal education, consideration of sex and age differences during child feeding practices.
